# Bilateral Cytomegalovirus Retinitis With Co-infection of Epstein-Barr Virus and Varicella-Zoster Virus: A Rare Case

**DOI:** 10.7759/cureus.99893

**Published:** 2025-12-22

**Authors:** Hirotaka Kondo, Mariko Egawa, Ryoji Yanai, Yoshinori Mitamura

**Affiliations:** 1 Department of Ophthalmology, Institute of Biomedical Sciences, Tokushima University Graduate School, Tokushima, JPN

**Keywords:** cytomegalovirus retinitis, epstein-barr virus infection, infectious uveitis, outcome of co-infection, uveitis, varicella-zoster virus infection

## Abstract

Cytomegalovirus retinitis (CMVR) is a form of infectious uveitis, a disease characterized by inflammation of the uvea, the middle layer of the eye. It is caused by the cytomegalovirus (CMV). Although CMVR generally involves a single virus, rare cases have been reported in which multiple viral DNAs have been detected in intraocular fluid using polymerase chain reaction (PCR) testing. The present case report documents a rare case of bilateral infectious uveitis with simultaneous detection of CMV and Epstein-Barr virus (EBV) in the right eye and CMV and varicella-zoster virus (VZV) in the left eye. A 75-year-old female patient with a history of long-term immunosuppressive therapy for rheumatoid arthritis presented with bilateral mild iritis and worsening retinal inflammation in the left eye. Fundus examination revealed granular exudates and retinal vascular occlusion in both eyes, with circumferential retinal lesions in the left eye. Qualitative PCR testing of the aqueous humor identified CMV and EBV in the right eye and CMV and VZV in the left eye. The patient was treated with intravenous acyclovir and ganciclovir, followed by intravitreal ganciclovir injections. Despite stabilization of the right eye, the left eye showed rapid lesion progression and developed a rhegmatogenous retinal detachment requiring surgical intervention. At the final follow-up, the corrected decimal visual acuity was 0.7 in the right eye and 0.02 in the left eye.Co-infection with multiple herpes viruses in viral retinitis is extremely rare. Immunosuppression may predispose individuals to such infections. PCR testing of intraocular fluid has been instrumental in diagnosis and guiding treatment. However, comprehensive evaluation is required to determine the pathogenicity of the viruses detected. This case highlights the importance of PCR testing for accurate diagnosis of atypical infectious uveitis. Although rare, concurrent infections with multiple herpesviruses may occur, and coinfection with VZV in particular could be associated with a poorer prognosis.

## Introduction

Cytomegalovirus retinitis (CMVR) is a type of infectious uveitis caused by cytomegalovirus (CMV) [1]. It predominantly occurs in individuals with severe immunosuppression, such as elderly patients, those with acquired immunodeficiency syndrome (AIDS), those with hematological disorders, or post-organ transplantation. CMVR is an opportunistic infection resulting from the reactivation of latent CMV.

While it is commonly observed in immunocompromised individuals in developed countries with aging populations, recent reports have also documented its occurrence in healthy individuals and those with mild immunosuppression [2, 3]. Othmanc et al. reported bilateral intermediate uveitis in cases of ocular CMV infection in individuals with intact immune systems [4]. CMVR is clinically classified into two subtypes: the fulminant posterior pole type and the granular peripheral type. The fulminant posterior pole type, typically observed in severely immunosuppressed patients, is characterized by extensive retinal edema and necrosis with associated hemorrhage [1]. In contrast, the granular peripheral type, which is observed in patients with mild immunosuppression, is characterized by peripheral granular exudative lesions with relatively slow progression. Before the widespread availability of antiretroviral therapy, the incidence of retinal detachment in CMVR was reported to be as high as 33%. However, with the introduction of treatment, the rate has declined to 8.7% [5].

While CMVR is typically associated with a single virus, there have been rare reports of multiple viral DNAs detected in intraocular fluid using polymerase chain reaction (PCR) testing [6-10]. Qualitative PCR testing can determine the presence or absence of a virus, while quantitative PCR can estimate viral load, which may suggest potential pathogenicity. However, clinical findings must also be considered to establish an accurate diagnosis. When multiple viruses are identified, it is often difficult to determine whether all detected viruses are truly pathogenic. We report a rare case in which different viruses were detected in both eyes simultaneously (right eye, CMV+EBV; left eye, CMV+VZV) and diagnosed as infectious viral retinitis involving multiple viruses based on clinical findings.

## Case presentation

**Figure 1:**
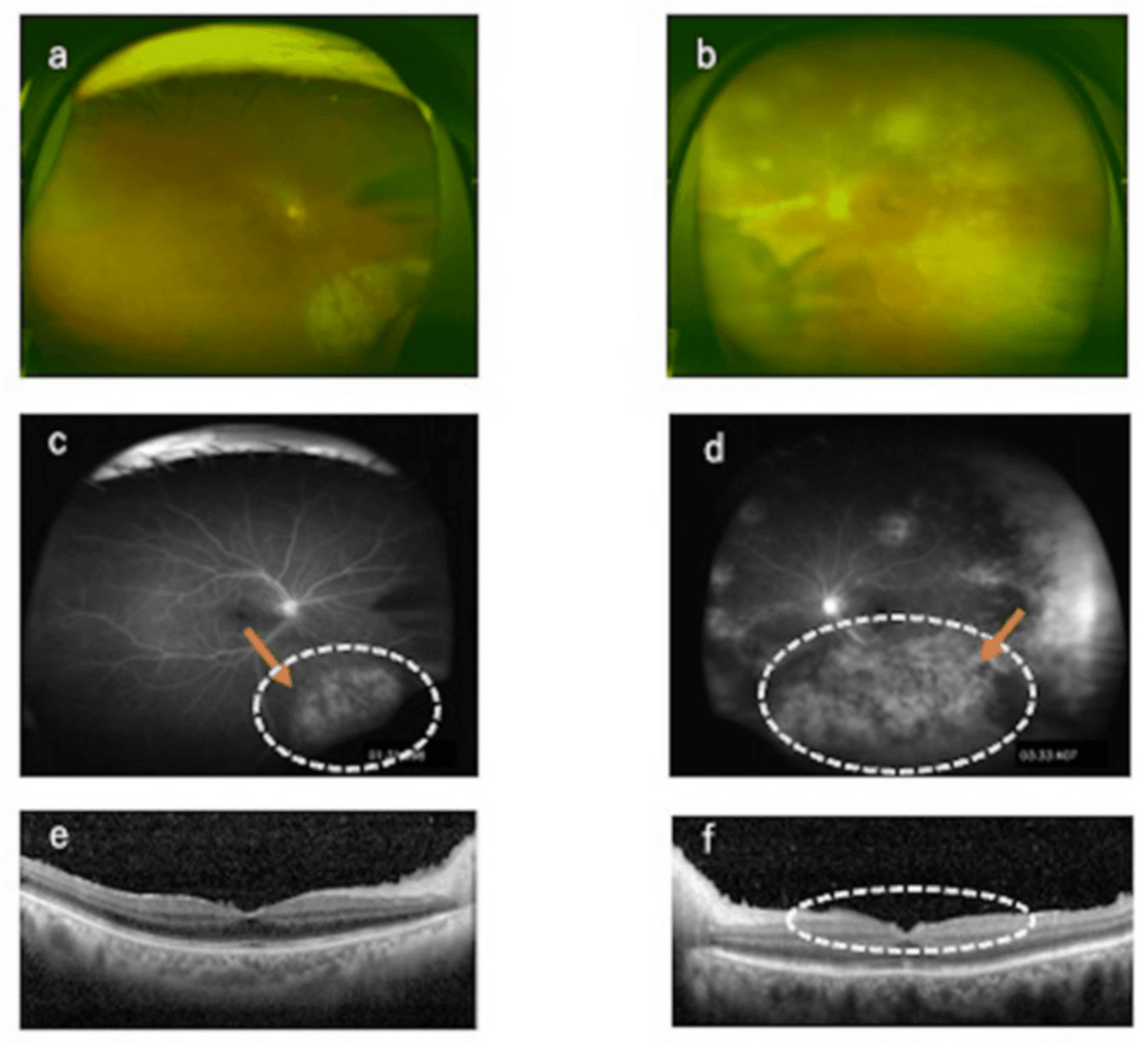
Fundus photographs, fluorescein angiography, and optical coherence tomography at initial presentation. (a) Right eye: Granular exudative lesions and retinal arterial sheathing observed in the inferonasal quadrant. (b) Left eye: Circumferential yellow-white granular lesions, coalesced exudative lesions, and retinal arterial sheathing and whitening were noted. (c) Right eye: Fluorescein leakage corresponding to the exudative lesions and staining of the retinal arterial walls. (d) Left eye: Retinal arterial wall staining, extensive vascular occlusion in the inferior retina, and optic disc hyperfluorescence observed. (e) Right eye: There were no abnormal findings in the macula. (f) Left eye: Mild hyperreflectivity in the inner retinal layers above the macula.

A 75-year-old female patient was referred to our hospital with 2+ anterior chamber cells in both eyes, according to the Standardization of Uveitis Nomenclature (SUN) grading criteria [1], worsening retinal inflammation in the left eye. Her medical history included type 2 diabetes mellitus and rheumatoid arthritis, for which she had been taking prednisolone (2 mg/day) and methotrexate (8 mg/week) for approximately 20 years. Prior to the onset of symptoms, the patient underwent surgical intervention under general anesthesia for severe carotid artery stenosis but had no history of herpes zoster. At the initial visit, the corrected decimal visual acuity was 0.2 in both eyes. Intraocular pressure measurements were recorded at 11 mmHg in the right eye and 10 mmHg in the left eye. A thorough fundus examination revealed the presence of coalescent and enlarged granular exudates accompanied by retinal artery sheathing in the inferonasal quadrant of the right eye. In the left eye, circumferential yellow-white granular lesions were observed, along with coalescent and enlarged yellow-white lesions, arterial sheathing, and whitening (Figures 1a, 1b).

Fluorescein angiography (FA) revealed optic disc hyperfluorescence in both eyes, with granular hyperfluorescence corresponding to exudates in the right eye and retinal arterial occlusion in the left eye. The left eye exhibited vascular wall staining, hyperfluorescence, and leakage in necrotic areas, widespread vascular occlusion, and avascular zones (Figures 1c, 1d). Optical coherence tomography (OCT) (SPECTRALIS®, Heidelberg Engineering, Heidelberg, Germany) revealed hyperreflective inner retinal layers above the macula in the left eye, consistent with retinitis (Figures 1e, 1f). Laboratory tests showed a normal white blood cell (WBC) count of 6,500/μL but a significantly reduced lymphocyte count of 180/μL. Although β-D-glucan levels were elevated, blood cultures were negative, and ocular findings ruled out fungal endophthalmitis. There was no evidence of tuberculosis or syphilis infection, as shown in Table 1. Aqueous humor samples from both eyes were collected and submitted for qualitative PCR testing at the time of the first visit to our hospital.

**Table 1:** Laboratory tests at initial visit CMV, cytomegalovirus; Hb, hemoglobin; PLT, platelet; RBC, red blood cell; VZV, varicella-zoster virus; WBC, white blood cell

Parameter	Result	Normal value
WBC	6500/µL	3,500-9,100/µL
Lymphocyte (rate)	2.8%	18-59%
Lymphocyte	180/µL	630-5,400/µL
RBC	3.21×10^6^/µL	3.76-5.00×10^6^
Hb	10.6 g/dL	11.3-15.2 g/dL
PLT	292×10^3^/µL	130-369×10^3^
CMV antigen	+2	(-)
CMV IgG	(+)	(-)
CMV IgM	(-)	(-)
VZV IgG	(+)	(-)
VZV IgM	(-)	(-)

Findings in the right eye suggested CMV retinitis localized to the periphery. The left eye showed marked vitreous opacity and widespread retinitis involving the macula, which rapidly deteriorated. Based on the clinical presentation, acute retinal necrosis was strongly suspected, prompting hospitalization and initiation of intravenous acyclovir (10 mg/kg every 8 h) and oral prednisolone (30 mg/day) until the PCR test results were available. The dosage of acyclovir was adjusted based on renal function and body size. On the fifth day of treatment, vitreous opacity in the left eye worsened, and the retinitis expanded to involve the macula; therefore, an intravitreal ganciclovir injection (500 mg) was administered to the left eye. By day 7, serum CMV pp65 antigen (C7-HRP) test result was positive, and the qualitative PCR test performed at the initial visit identified Epstein-Barr virus (EBV) and CMV in the aqueous humor of the right eye and varicella-zoster virus (VZV) and CMV in the aqueous humor of the left eye, as shown in Table 2. Subsequently, intravenous ganciclovir (2.5 mg/kg every 24 h) was added to the treatment regimen, guided by creatinine clearance [11]. On the 10th day, intravenous acyclovir was switched to oral valacyclovir at a dosage of 3,000 mg per day. Following systemic administration of ganciclovir, the necrotic lesion in the left eye showed no further progression; however, vitreous opacity continued to worsen. Subsequently, a rhegmatogenous retinal detachment developed in the left eye, necessitating a 25-gauge pars plana vitrectomy accompanied by cataract surgery, encircling scleral band placement, and silicone oil tamponade. The quantitative real-time PCR of the vitreous humor revealed 6.7 × 10^7^ copies/mL of CMV and 1.3 × 10^8^ copies/mL of VZV (Table 3).

**Table 2:** Qualitative PCR testing (aqueous humor) at initial visit CMV, cytomegalovirus; EBV, Epstein-Barr virus; HSV, herpes simplex virus; L, left; PCR, polymerase chain reaction; R, right; Toxo, toxoplasma; TP, Treponema pallidum; (-), negative; (+), positive

Parameter	Result	Normal value
HSV-1 (R)	(-)	(-)
HSV-1 (L)	(-)	(-)
HSV-2 (R)	(-)	(-)
HSV-2 (L)	(-)	(-)
VZV (R)	(-)	(-)
VZV (L)	(+)	(-)
EBV (R)	(+)	(-)
EBV (L)	(-)	(-)
CMV (R)	(+)	(-)
CMV (L)	(+)	(-)
HSV-6 (R)	(-)	(-)
HSV-6 (L)	(-)	(-)
HTLV-1 (R)	(-)	(-)
HTLV-1(L)	(-)	(-)
Toxo (R)	(-)	(-)
Toxo (L)	(-)	(-)
TP (R)	(-)	(-)
TP (L)	(-)	(-)

**Table 3:** Quantitative real-time PCR testing (vitreous humor) in the left eye CMV, cytomegalovirus; VZV, varicella-zoster virus; (-), negative; (+), positive

Parameter	Result	Normal value
CMV	(+) (6.7×10^7^)	(-)
VZV	(+) (1.3×10^8^)	(-)

Following the initiation of intravenous ganciclovir, the granular white exudates in the inferonasal quadrant of the right eye resolved, and the serum CMV antigen became negative. On day 17, intravenous ganciclovir was switched to oral valganciclovir, which was discontinued on day 38 due to a deterioration in renal function. Although iritis and retinitis subsided, a white exudative lesion appeared near the optic disc in the right eye on day 81. Laboratory tests showed positive CMV antigenemia and reduced lymphocyte count (530/μL). Aqueous humor PCR detected 2.6 × 10^3^ copies/mL of CMV, leading to a diagnosis of recurrent CMVR. Oral valganciclovir (450 mg) was restarted, resulting in inflammation reduction. Valacyclovir and prednisolone were tapered and discontinued over a period of six months.

**Figure 2:**
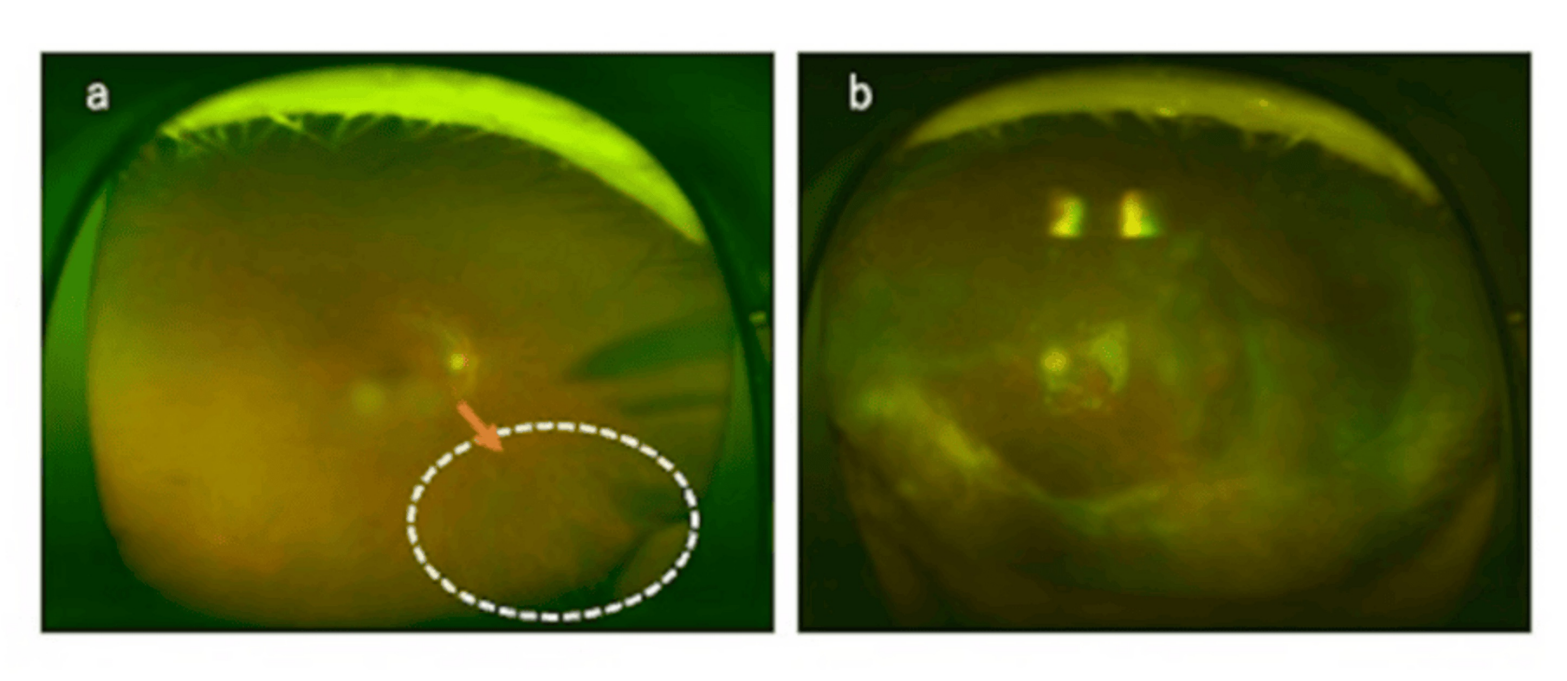
Fundus photographs eight months after onset. (a) Right eye: Resolution of inferonasal exudative lesions and vasculitis, leaving atrophic scars. (b) Left eye: After vitrectomy surgery, proliferative membrane formation on the inferior retina and persistent retinal detachment remained.

At the final follow-up, eight months after the onset of symptoms, the corrected visual acuity was 0.7 in the right eye and 0.02 in the left eye. The right eye exhibited residual atrophic scarring at the lesion site, while the left eye demonstrated persistent macular edema, proliferative membrane formation, and retinal detachment in the inferior retina (Figures 2a, 2b).

## Discussion

We present a rare instance of bilateral infectious uveitis, characterized by the concurrent detection of CMV and EBV in the right eye and CMV and VZV in the left eye. While the simultaneous detection of multiple viruses in viral retinitis is infrequent, previous studies have indicated that elderly individuals with mild immunosuppression may develop retinitis caused by multiple herpes viruses [9]. A summary of previous reports on multiple-virus detection is provided in Table 4 [6-10].

**Table 4:** Reports of multiple viral retinitis detected by PCR AIDS, acquired immune-deficiency syndrome; B, both; CMV, cytomegalovirus; CMVR, cytomegalovirus retinitis; EBV, Epstein-Barr virus; F, female; HSV, herpes simplex virus; IV, intravenous injection; L, left; M, male; PCR, polymerase chain reaction; PORN, progressive outer retinal necrosis; R, right; VZV, varicella-zoster virus; VI, intravitreal injection

Authors	Case	Diagnosis	Primary illness	Diagnostic sampling	Virus detected by PCR	Copy number of PCR (copies/mL)	Treatment (Local)	Treatment (Systemic)
Lopez et al., 2024 [6]	64 M	CMVR (R)	Burkitt lymphoma	Aqueous humor	CMV+HSV+VZV	N/A	Foscarnet VI	Oral valganciclovir
Sato et al, 2018 [10]	75 F	Chronic uveitis (L)	Rheumatoid arthritis	Vitreous fluid	EBV+VZV	EBV: 6×10^5^; VZV: 4×10^6^	Betamethasone eye drops	Oral albendazole; oral corticosteroid
Samanta et al., 2021 [7]	32 F	CMVR (R)	Pemphigus vulgaris	Aqueous humor	CMV+HSV-1	N/A	Ganciclovir VI	Acyclovir IV
Liu et al., 2024 [8]	39 F	CMVR (B)	Acute lymphoblastic leukemia	Aqueous humor	CMV+EBV+HSV(R), CMV(L)	N/A	Ganciclovir VI	N/A
Moharana et al., 2020 [9]	17 M	PORN (L)	AIDS	Vitreous fluid	CMV+VZV	CMV: 4×10^5^; VZV: 5×10^4^	Ganciclovir Vl	Oral valaciclovir
Our case	75 F	CMVR (B)	Rheumatoid arthritis	Vitreous fluid	CMV+VZV	CMV: 6.7×10^7^; VZV: 1.3×10^8^	Ganciclovir Vl	Acyclovir IV; ganciclovir IV

To our knowledge, no particular condition has been reported for bilateral co-viral multiple infectious uveitis. Some studies showed some cases of co-viral infectious uveitis. Samanta et al. reported a case of CMVR with simultaneous detection of CMV and HSV in the same eye during immunosuppressive therapy [7]. The case presented with atypical CMVR, characterized by severe inflammation and retinal vascular occlusion. The authors concluded that the coinfection of CMV and HSV contributed to the pathology and reported that combining local ganciclovir treatment with systemic acyclovir successfully resolved the retinitis. Liu et al. described a case of bilateral CMVR in which CMV was detected in the left eye and CMV, EBV, and HSV in the right eye [8]. The patient had undergone hematopoietic stem cell transplantation and was in a state of profound immunosuppression. Although three viruses were detected in the right eye, the retinal lesions were limited to peripheral yellow-white exudates with hemorrhage. The authors hypothesized that EBV and HSV were non-pathogenic and reported successful treatment with intravitreal ganciclovir monotherapy. In the report by Liu et al., three viruses, including EBV, were simultaneously detected; however, not all were considered causative agents of retinitis. They concluded that the role of EBV in causing retinitis in immunocompromised patients remains debatable. In infectious uveitis caused by multiple viruses, overlapping clinical findings can make diagnosis challenging based solely on ocular examination. Therefore, PCR testing of intraocular fluid for viral identification is invaluable for diagnosis and treatment planning. However, determining the pathogenicity of detected viruses and identifying the causative virus for retinitis require a comprehensive evaluation, including ocular findings, the patient's immunological status, and response to antiviral therapy. In our case as well, EBV was detected; however, it was not possible to determine whether it was truly the causative pathogen.

In our case, immunosuppression resulting from long-term immunosuppressive therapy for rheumatoid arthritis, in conjunction with stress following vascular surgery, likely contributed to the development of bilateral CMVR with multiple-virus infection. According to the results of PCR testing of the aqueous humor, combined acyclovir and ganciclovir therapy was initiated promptly. Consequently, CMVR in the right eye stabilized without further progression, and visual acuity was preserved. In contrast, the left eye showed rapid lesion expansion within a short period, resulting in rhegmatogenous retinal detachment and poor visual prognosis. The coexistence of VZV infection may have contributed to the exacerbation of inflammation. Calvo et al. reported that patients with viral DNA copy numbers ≥5.0×10⁶ copies/mL in the aqueous humor also had a higher incidence of retinal detachment [12]. In our case, both CMV and VZV showed high copy numbers in the vitreous fluid collected during surgery, indicating a high risk of retinal detachment. It is important to perform quantitative PCR from the initial examination and consider early surgery before retinal detachment occurs if high copy numbers are detected.

This case report has some limitations. First, PCR testing was not performed at the referring institution when the uveitis first developed, leaving the timing and mechanism of the multiple-virus infection unclear. Secondly, the initial aqueous humor PCR was qualitative, precluding the determination of viral copy numbers of CMV, VZV, and EBV. However, quantitative PCR of the vitreous humor later revealed high CMV and VZV viral loads, suggesting their pathogenicity. Nonetheless, as quantitative testing for EBV was not performed, its contribution to inflammation was deemed minimal based on clinical findings and disease course. In future cases of atypical retinitis with diagnostic uncertainty, quantitative PCR testing should be considered.

## Conclusions

Although extremely rare, simultaneous infections involving distinct herpesviruses in both eyes can occur and may cause pathogenic, inflammatory responses. For suspected cases of infectious uveitis, PCR testing of intraocular fluid is essential for accurate diagnosis and determining the treatment strategy. Furthermore, in atypical cases where multiple viruses are detected, quantitative PCR is useful for assessing viral activity. Ultimately, the pathogenicity must be determined based on both the clinical course and the PCR results. In cases of rapidly progressive retinitis, early surgical intervention, such as vitrectomy, should be considered to preserve visual function.
